# Supplementary material for: Prognostic Significance of Lymphocyte Infiltration and a Stromal Immunostaining of a Bladder Cancer Associated Diagnostic Panel in Urothelial Carcinoma
Source: Diagnostics (Basel). 2019 Dec 28;10(1):14. doi: 10.3390/diagnostics10010014 (PMC7168167; doi:10.3390/diagnostics10010014)
Supplement: Supplementary file 1 [file diagnostics-10-00014-s001.pdf]

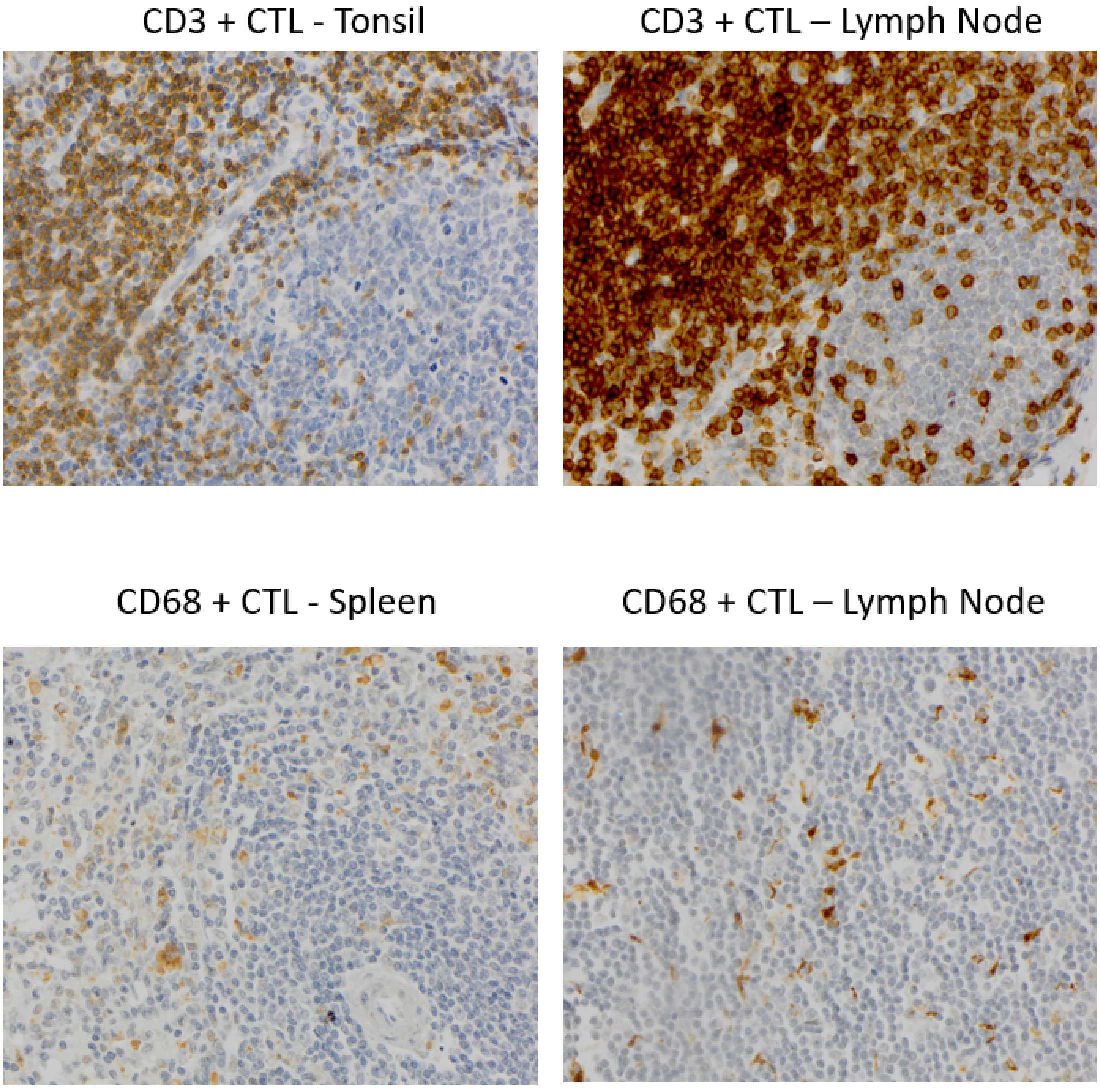

Supplemental Figure S1. Progression-free survival analysis of 213 patients with bladder cancer. Progression-free survival according to immunostaining status of > 5 targets from diagnostic signature, 3-5 targets from diagnostic signature and < 3 targets from diagnostic signature with or without infiltration of CD3+ and CD68+ cells.
